# The Fourth Annual Meeting of the National Dental Association, Held at Milwaukee, Wis., Aug. 6–9, 1901

**Published:** 1901-09-15

**Authors:** 


### THE FOURTH ANNUAL MEETING OF THE NATIONAL DENTAL ASSOCIATION, HELD AT MILWAUKEE, WIS., AUG. 6-9, 1901.

At half-past eleven, Tuesday morning, President Black called the Association to order, and Dr. Noyes, of Chicago, invoked Divine blessing upon the proceedings.

Dr. G. V. I. Brown, on behalf of the Mayor of the city, extended the official hospitality of the city. He also, for the dental profession, announced that certain social functions had been prepared and would be announced later to harmonize with the Association's program. He also announced two tally-ho coach rides for the ladies each day, and expressed the hope that every one might feel perfectly free to ask for anything he might want, and which he could not find on the regular program.

Dr. C. N. Johnson, of Chicago, in fitting terms accepted the cordial welcome ; he said it was unusual for this Association to be entertained, but knowing the hospitable character of our hosts he was not surprised at the well-laid plans for our entertainment. He expressed the hope that we might not in all this gaiety loose sight of the object for which we had assembled, and that each would do what he could to assure a successful meeting.

President Dr. G. V. Black then read his address. He briefly reviewed the work of the National Dental

Association from its organization in 1860 up through its several organizations to the present time, and called attention to the wonderful progress made by the profession and the part taken by the National organization as the controlling influence in shaping and carrying forward high professional standards.

He also took occasion to analyze some of the conditions at present prevailing in the Association, first calling attention to the membership. Originally it was intended that the National organization should be a representative body, and that its meetings should be largely attended and constituted by delegates from the various State, district and local organizations. He called attention to the fact that in 1899 the National Dental Association, instead of being largely a delegate body, had a permanent membership of 369 and a delegate representation from only twenty organized societies of all kinds, indicating that the original purpose had been lost sight of. This he deplored and suggested that steps be taken to secure a representation from every local, district and State organization in the country. In order that this object might be secured, at least so far as State representation is concerned, he proposed that the constitution be amended so that all permanent memberships shall lapse in any State which fails to send a delegate for two consecutive years. He also suggested that a quickened interest in the proceedings of the National organization might be secured by distributing a copy of the Association's proceedings to each member of all State associations represented.

He also brought out the fact that the papers read at meetings of this organization were too often what might be classed as "literature of the day," rather than the result of scientific research. Such papers are interesting when well written, but add little to the resources of our profession. It has come to be that the best scientific papers are read before the more select dental societies, where they will receive adequate discussion or published in the better journals where they will have a wide reading, rather than to be buried in our proceedings for a year before they can be seen by the profession. And as our profession can not as yet be characterized as a reading one these papers are likely to have little influence.

Another difficulty which the association had encountered, was that too large a number of papers had been offered in the past and even at the present meeting to make it possible to give each one adequate consideration. To overcome this difficulty he recommended that the section plan as used in the American Medical Association be adopted. By having three sections before which appropriate papers could be simultaneously discussed, and general meetings for executive work only, this congestion might be avoided and each subject would be carefully considered by those most interested, and three times as much work accomplished in the same time as now.

The address also advised a radical revision of the constitution, to the end that executive business might be transacted by the executive officers only and thus the time of the general meeting be conserved for the literary program.

The time of the year in which the meetings are held was also a very important matter to consider. The summer is not most favorable for work. The middle of September or last of February would seem to be much more favorable times for holding meetings.

The address expressed the hope that the time would soon come when all the dental organizations of a national character might be federated. And the National Examiners, Faculties and Pedagogic Associations might meet at the same time as the National Dental Association. This would be more economical in time and money for all who might desire to attend the proceeding of these several organizations, and the proceedings would broaden the outlook of all.

---

Dr. Finley reported the successful conclusion of the work of the committee appointed to secure legislative enactment providing for dental surgeons in the Army. He reported that the complement of thirty army dental surgeons were mostly under appointment and some were at work on the field, and that by fall all would be at work. The examining board had taken such steps as would likely provide only the best men for the service.

